# Dual exposure to smoking and household air pollution is associated with an increased risk of severe asthma in adults in Brazil

**DOI:** 10.1186/s13601-018-0235-6

**Published:** 2018-12-10

**Authors:** Andréia Guedes Oliva Fernandes, Carolina de Souza-Machado, Gabriela Pimentel Pinheiro, Sergio Telles de Oliva, Raquel Cristina Lins Mota, Valmar Bião de Lima, Constança Sampaio Cruz, José Miguel Chatkin, Álvaro A. Cruz

**Affiliations:** 1 Programa para o Controle da Asma na Bahia (ProAR) e Programa de Pós-graduação em Medicina e Saúde, Centro de Saúde Carlos Gomes, Faculdade de Medicina da Bahia, Universidade Federal da Bahia (UFBA), Rua Carlos Gomes 270, 7° andar, Salvador, Bahia 40060-330 Brazil; 2 Programa para o Controle da Asma na Bahia (ProAR) e Programa de Pós-graduação em Medicina e Saúde, Faculdade de Medicina da Bahia, Escola de Enfermagem da Universidade Federal da Bahia, UFBA, Rua Dr Augusto Vianna. 2 andar. Campus Canela, Salvador, Bahia 40110-060 Brazil; 3 Laboratório de Química Analítica Ambiental, Departamento de Química Analítica, Instituto de Química, Universidade Federal da Bahia, Brazil, Rua Barão de Jeremoabo, 147. Campus de Ondina, Salvador, Bahia 40170-115 Brazil; 4 Serviço de Endoscopia Digestiva do Hospital das Clínicas de Universidade de São Paulo, PAMB, Prédio dos Ambulatórios, Av. Dr. Enéas Carvalho de Aguiar, 155 - 6°. Andar, Sala 3, São Paulo, SP CEP 05403-900 Brazil; 5 Programa para o Controle da Asma na Bahia (ProAR), UFBA - Centro de Saúde Carlos Gomes, Rua Carlos Gomes 270, 7° andar, Salvador, Bahia 40060-330 Brazil; 6 Programa de Pós-Graduação em Medicina e Saúde Humana. Escola Bahiana de Medicina e Saúde Pública (EBMSP), Obras Sociais Irmã Dulce. Hospital Santo Antônio - Avenida Bonfim, 161 Largo de Roma, Salvador, Bahia 40420-415 Brazil; 7 Escola de Medicina da Pontifícia, Universidade Católica do Rio Grande do Sul, Brazil, Av. Ipiranga, 6690, Porto Alegre, Rio Grande do Sul 90610-000 Brazil

**Keywords:** Asthma, Control, Severity, Air pollution indoors, Smoking

## Abstract

**Background:**

The relationship between smoking, household pollution, dual exposure and severity of asthma in adults has not been sufficiently studied. We examined and compared the effects of cigarette smoking, domestic wood burning pollution and dual exposure (tobacco and wood burning) upon asthma severity in adults.

**Methods:**

This was a cross-sectional study performed with 452 individuals with mild to moderate asthma and 544 patients with severe asthma (previously untreated). Smoking and exposure to wood smoke were identified and quantified through questionnaires to evaluate current and/or previous exposure; objective determination of cigarette exposure was obtained through the measurement of urinary cotinine. Asthma control was evaluated through Asthma Control Questionnaire; and severity was classified according to the Global Initiative for Asthma criteria. Subjects were grouped according to exposure type into 4 groups: smokers, household pollution, dual-exposure and no-exposure. Chi square, Mann–Whitney, and Kruskal–Wallis tests were used for comparisons between groups.

**Results:**

Out of 996 included individuals, 78 (7.8%) were exposed to cigarette smoking alone, 358 (35.9%) to household pollution alone, 155 (15.6%) to the two exposures combined and 405 (40.7%) were not exposed. Compared to unexposed individuals, exposure to household pollution resulted in poorer asthma control, higher proportion of severe asthma, and worse indicators of lung function. The double-exposed individuals were worse off in all the evaluated parameters, and they were significantly worse than subjects with single exposure to household air pollution in relation to asthma severity and lung function. These subjects were predominantly females, older, with longer residence time in rural areas, lower income and lower schooling levels. Multivariate analysis showed that exposure to household pollution and double exposure were predictive factors associated with lack of control and increased severity of asthma.

**Conclusions:**

Exposure to household pollution is associated with poorer control, greater severity, and poorer pulmonary function; double-exposed individuals have a greater risk of severe asthma and decreased lung function than those exposed only to household pollution.

## Background

Asthma is a chronic respiratory disease affecting some 334 million people worldwide [1]. Its prevalence in adults varies from 1 to 21% [2, 3]. In the last decades, the asthma epidemic may have reached its peak and some evidence suggests a steady increase in this prevalence in some countries, while others have shown a reduction in recent years and these changes are the result of the interaction of various factors in the environment [4]. People with asthma are more sensitive to the effects of respiratory irritants such as environmental pollutants and smoke, because of the hyper-responsiveness of the airways. Exposure to smoke or environmental pollutants may result in increased airway inflammation and worse clinical outcomes [5–7]; such exposures increases morbidity and mortality in the general population, and most particularly in susceptible subgroups such as asthmatics [8].

Products generated by firewood and cigarette combustion contribute to air pollution, particularly in indoor environments; they represent an avoidable risk factor which worsens the burden of respiratory diseases [9, 10]. Several studies suggest that smoking or exposure to second hand cigarette smoke result in greater asthma severity, accelerated decline in lung function, reduced response to inhaled corticosteroids, increased use of health services, and poorer asthma control [7, 11–14]. Household exposure to smoke of firewood is also associated with the risk of asthma and respiratory symptoms [10, 15, 16].

Exposure to household air pollution is related to a dependence upon biomass as a fuel for daily activities; its use is typical of rural areas and frequent among people in lower income settings [17, 18]. Smoking is a public health problem affecting all social classes and responsible for 6 million preventable deaths/year, worldwide [19]. In many scenarios of ordinary life, exposures to these agents may overlap, but few studies have evaluated such dual exposure afflicting asthmatic adults. Thus, the objective of this study was to evaluate the effects of household pollution by exposure to wood stove smoke and smoking, either alone or in combination, on the control and severity of asthma in adults in Brazil.

## Methods

### Study design and setting

The study was performed in a reference centre for severe asthma of Program for Asthma Control (ProAR) in the Brazilian State of Bahia. This is a preliminary cross-sectional analysis of data collected for a case–control study of risk factors for severe asthma, which includes cases from the ProAR Cohort of severe asthma, enrolled from 2003 according a previous definition of Global Initiative for Asthma [20] and two control groups: (i) participants with mild to moderate asthma (according to the 2012 GINA classification) [21]; (ii) persons with no asthma. Only the patients with asthma were included in this preliminary analysis and report. Subjects enrolled in the ProAR Cohort of severe asthma, at the time of admission, would match the criteria of untreated severe asthma proposed to the World Health Organization in 2009 [22]. The ProAR program encompasses prevention and care actions carried out by a multi-professional team caring for patients with severe asthma within the Brazilian Unified Health System (SUS), in order to achieve and maintain disease control [23].

### Sample selection

Participants with severe asthma were recruited from the ProAR cohort. Patients with mild to moderate asthma were recruited within the community through publicity in the media, in public areas, in public transport and other. Potential study participants were contacted by phone for a pre-screening, at which point they were assessed for inclusion criteria and then invited to attend the research clinic.

Inclusion criteria were: (i) being an user of the Brazilian Unified Health System (SUS) with a medical diagnosis of asthma, (ii) age ≥ 18 years, (iii) residence in Salvador, Bahia, or in its metropolitan region. Exclusion criteria were: (i) patients with any comorbidities that prevented an accurate evaluation of asthma control (congestive heart failure, stroke, myopathies, advanced neoplasia, psychiatric illnesses or other lung diseases); (ii) patients previously reporting a history of smoking > 10 pack/years (in a screening process), due to the difficulty of differential diagnosis with Chronic Obstructive Pulmonary Disease (COPD). A total of 996 asthmatic patients were included, 452 with mild to moderate asthma, 544 with severe asthma, the latter being followed up in ProAR for at least 6 months.

Validation of the diagnosis of asthma among the patients of the severe asthma cohort was performed by two medical specialists through a review of their clinical records and complementary exams, including spirometry and chest X-Ray. A standardized report was independently completed by each physician. Report options were: (A) The patient has asthma confirmed by spirometry with reversible obstruction; (B) The patient probably has asthma by the typical history, but has no reversible obstruction observed; (C) The patient may have asthma, but the condition is not typical nor there is reversible obstruction; (D) The patient has no asthma. Only patients categorized by both specialists as options A or B were included in the study. Discordant diagnoses were submitted to a third specialist. Subjects of the severe asthma group B have a medical diagnosis of asthma based on a typical pattern of symptoms and response to treatment. Their charts were reviewed and the diagnosis was validated by two independent specialists. The studies have been conducted in a secondary health care facility, where bronchoprovocation is unavailable.

The patients with severe asthma come from the ProAR cohort, where they were receiving regular treatment for at least six months. Their provision of care and medication is maintained in our secondary care reference center. Conversely, patients with mild-to-moderate asthma were recruited from the community and were interviewed and evaluated only once. At the time of the interview they were asked about the use of medications in the last three months. Most of these subjects were not receiving regular treatment.

### Data collection

All participants were evaluated by a specialist at the study visit to check the inclusion and exclusion criteria, classification of asthma control according to GINA [21], collection of sociodemographic, clinical, spirometric and exposure information.

Trained interviewers applied the Asthma Control Questionnaire in the six-questions version (ACQ-6), a questionnaire validated for Brazilian Portuguese [24] assessing asthma symptoms and use of bronchodilators for relief in the last seven days. We adopted the score ≥ 1.5 as an indicator of uncontrolled asthma [24, 25].

All participants eligible for the study were submitted to spirometry for measurements of Forced Expiratory Volume in the first second (FEV_1_), FEV_1_ to Forced Vital Capacity (FVC) ratio (FEV_1_/FVC) and Forced Expiratory Flow 25–75% (FEF_25–75%_) before and 15 min after inhaling 400 mcg of salbutamol, as recommended by the American Thoracic Society/European Respiratory Society (ATS/ERS) [26] using a Koko^®^ Spirometer (Ferraris Medical, USA).

### Evaluation of smoking

A patient was considered smoker, if he/she self-declared him/herself to be a current or previous smoker. Previous smoking was defined by self-reported cessation of smoking for at least six months. The following question was posed during the initial interview: current and previous smokers were asked to provide the average number of cigarettes per day and the number of smoking years; the smoking load (pack/years) was calculated by the physician.

Information regarding exposure to second hand cigarette smoke was assessed by applying the questionnaire adapted from the Brazilian Institute of Geography and Statistics (IBGE) used in the 2010 Census [27]; which was divided into three domains: home exposure, exposure in the school and/or work environment and exposure in transportation and public environments. The criteria used to define individuals with exposure to second hand cigarette smoke were: presence of occupational exposure and/or home exposure. The application of the questionnaires was performed by a team of trained interviewers.

Patients were stratified for smoking load as < 10 pack/years or ≥ 10 pack/years. In addition, exposure to smoking was also objectively evaluated, through the quantification of urinary cotinine, the standard biomarker of exposure to nicotine. To determine urinary cotinine concentrations, morning urine samples were collected at the time of the interview using sterile vials. The samples were preserved under refrigeration (− 70 °C) for future laboratory analysis. Measurement of urinary cotinine was performed according to a procedure described by Cattaneo et al. [28]. An Agilent^®^ Infinity 1290 high-performance liquid chromatograph was used, with a Zorbax Eclipse XDB-C8 (4.6 mm × 150 mm × 5 μm) and UV–Vis detector (μ = 260 nm), injection volume of 20 μL and mobile phase flow rate of 0.4 mL.min^−1^. The validation of the methodology was performed according to the parameters described by Resolution 899 of the Brazilian National Agency of Sanitary Surveillance (ANVISA) [29]. To improve the accuracy of cotinine measurement, its value was corrected for urinary creatinine, thus obtaining the urinary cotinine/creatinine ratio expressed in μg/g, and the biochemical confirmation of the smoking status was considered when the urinary cotinine values ≥ 196.98 μg/g [30], a cutoff point providing the best accuracy in our sample. Urinary creatinine was quantified using a creatinine K assay kit using a biochemical automation system BT 3000 PLUS (Wiener Lab Group, Argentina).

### Evaluation of exposure to household pollution by firewood combustion smoke

Exposure to the smoke of the wood stoves was deemed to occur whenever an individual reported current and/or previous exposure. A previous history of exposure to combustion of firewood was established through self-reported exposure and cessation. Assessment was made at the time of the interview conducted by the physician, who investigated the following items: presence of current and/or prior exposure, number of years of exposure. Participants were stratified for exposure time < 10 years and ≥ 10 years [31].

### Statistical analysis

The sample size of 500 in each group was calculated for objectives of the larger case–control study, taking into consideration several potential major risk factors. For the study reported herein we assume this is a convenience sample size. Data were analysed using the Statistical Package for Social Science (SPSS) version 17.0. Categorical variables were expressed as absolute frequency and percentages; continuous variables were expressed as mean and standard deviation, or median and the interquartile range (percentile 25 to percentile 75), depending on the nature of the variable. Chi square, Mann–Whitney and Kruskal–Wallis tests were performed for comparisons between groups depending on the nature of the variable. The factors associated with asthma control and severity were inserted into the multiple logistic regression model.

### Study approval

The study was approved by the Ethics and Research Committee of the Federal University of Bahia (case # 099/2009, additive # 032/2014), as well as by the National Commission for Ethics in Research (case # 450/2010). All participant patients read and signed a written informed consent.

## Results

### General

Of the 996 patients evaluated, 78 (7.8%) were exposed only to cigarette smoking, 358 (35.9%) only to household air pollution, 155 (15.6%) were doubly exposed, to smoking and household pollution, while 405 (40.7%) subjects denied either exposure. Table 1 displays the sociodemographic characteristics of the sample. A predominance of females was observed in all groups. The dual-exposure group had the highest mean age (54 ± 12 years), while the non-exposure group presented the lowest (38 ± 14 years). The dual-exposure group had lower income, lower educational level and longer residence time in rural areas compared to other groups.

**Table 1:** Sociodemographic characteristics of the individuals with asthma (mild to moderate and severe) studied

Characteristics	Smokers (n = 78)	Household pollution (n = 358)	Double exposure (n = 155)	No exposure (n = 405)	P^§^
Sex n (%)					
Female	49 (62.8)^a.b^	310 (86.6)^d.e^	115 (74.2)	321 (79.3)	< 0.001
Male	29 (37.2)	48 (13.4)	40 (25.8)	84 (20.7)	
Average age (years ± SD)	43 ± 14^a.b.c^	50 ± 14^d.e^	54 ± 12^f^	38 ± 14	< 0.001
Family income (BRL) M (P_25–75_)	1000 (724–1800.)^b.c^	800 (682– 1400)^e^	724 (678–1300)^f^	1100 (786–1800)	< 0.001
Marital status					
Single	44 (56.4)^b^	136 (38.0)^e^	66 (42.6)^f^	205 (50.6)	0.002
Married	19 (24.4)	115 (32.1)	38 (24.5)	109 (26.9)	
Other^†^	15 (19.2)	107 (29.9)	51 (32.9)	91 (22.5)	
Self-referred skin colour					
Mulatto	38 (48.7)	181 (50.6)	91 (58.7)^f^	185 (45.7)	0.150
Black	30 (38.5)	145 (40.5)	53 (34.2)	182 (44.9)	
Other^‡^	10 (12.8)	32 (8.9)	11 (7.1)	38 (9.4)	
Educational level					
None	01 (1.3)^b.c^	23 (6.4)^d.e^	13 (8.4)^f^	03 (0.7)	< 0.001
Primary	06 (7.7)	70 (19.6)	55 (35.5)	21 (5.2)	
2nd grade	13 (16.7)	90 (25.1)	42 (27.1)	47 (11.6)	
High school	40 (51.3)	148 (41.3)	40 (25.8)	219 (54.1)	
University or higher	18 (23.1)	27 (7.5)	05 (3.2)	114 (28.1)	
No information	–	–	–	01 (0.2)	
Has lived in rural area	18 (23.1)^b.c^	212 (59.2)^e^	94 (60.6)^f^	106 (26.2)	< 0.001
Years in rural areas M(P_25–75_)	12 (5–19)	14 (10–18)^e^	16 (11–20)^f^	12 (7–17)	0.003

M(P_25–75_): Median (Percentile 25–75), *BRL* Brazilian Reais

^a^*P *≤ 0.05 smokers versus unexposed

^b^*P *≤ 0.05 smokers versus domestic exposure

^c^*P *≤ 0.05 smokers versus double exposure

^d^*P *≤ 0.05 domestic exposure versus double exposure

^e^*P *≤ 0.05 domestic exposure versus no exposure

^f^*P *≤ 0.05 double exposure versus no exposure

^†^Others—stable union; divorced/separated; widowed

^‡^Others—White. Native American or Oriental

^§^*P* value for 4-group comparisons

### Exposure

The intensity of exposure to smoking (pack/years) and household pollution (years of exposure) were: (i) tobacco only: 64 (82.1%) exposed to < 10 pack/years and 14 (17.9%) exposed to ≥ 10 pack/years; (ii) household pollution only: 81 (22.6%) < 10 years exposure and 277 (77.4%) ≥ 10 years exposure; (c) dual-exposure to smoking and household pollution: 61 (39.4%) ≥ 10 pack/years and 132 (85.2%) ≥ 10 years of households pollution exposure. The dual-exposure group had the highest mean duration in years of exposures to tobacco (12 ± 18 pack years) and to firewood (16 ± 08 years); in comparison, the group exposed to cigarette smoking only averaged 05 ± 9 pack years, while the group exposed only to firewood averaged 14 ± 07 exposure/years.

The characteristics of the exposures are shown in Table 2. We found a statistically significant difference in the comparison between groups (p < 0.001). The current smoking frequency was higher in the group exposed to tobacco alone (16.7%), while previous smoking was more common in the dual exposure group (94.8%). In the group exposed to smoking alone, a higher frequency of secondary exposure to cigarette smoke was observed (30.8%). Exposure to cigarette smoke in the last 24 h reached 46.2%. Confirmation of smoking status was obtained through the urinary levels of the cotinine/creatinine ratio (> 196.98 μg/g), being significantly higher among patients with dual exposure, reflecting higher intensity of cigarette exposure in this subgroup. Patient’s information on smoking currently were concordant with the cotinine measurements.

**Table 2:** Characteristics of exposure of the individuals with asthma (mild to moderate and severe) studied

Characteristics	Smokers (n = 78)	Household air pollution (n = 358)	Double exposure (n = 155)	No exposure (n = 405)	*P* ^§^
	n (%)	n (%)	n (%)	n (%)	
Current smokers	13 (16.7)^a.b.c^	–	08 (5.2)^f^	–	< 0.001
Former smokers	65 (83.3)^a.b.c^	–	147 (94.8)^f^	–	< 0.001
Second hand tobacco exposure	24 (30.8)^b^	61 (17.0)	37 (23.9)	87 (21.5)	0.033
Domestic exposure to cigarette smoke	12 (15.4)	30 (8.4)^d^	26 (16.8)	44 (10.9)	0.029
Domestic exposure to cigarette smoke over last 24 h	06 (7.7)	11 (3.1)^d.e^	18 (11.6)^f^	26 (6.4)	0.003
Occupational exposure to cigarette smoke	14 (17.9)^b^	34 (9.5)	16 (10.3)	50 (12.3)	0.164
Occupational exposure to cigarette smoke over last 24 h	07 (9.0)	17 (4.7)	07 (4.5)	17 (4.2)	0.347
Cigarette smoke exposure over last 24 h	36 (46.2)^a.b^	105 (29.3)^d^	59 (38.1)	128 (31.6)	0.016
Urinary cotinine/creatinine ≥ 196.98 μg/g	06 (7.7)^a.b^	07 (2.0)^d^	18 (11.6)^f^	08 (2.0)	< 0.001
Present exposure to wood combustion smoke	–	06 (1.7)^e^	–	–	0.013

^a^*P *≤ 0.05 smokers versus unexposed

^b^*P *≤ 0.05 smokers versus domestic pollution

^c^*P *≤ 0.05 smokers versus double exposure

^d^*P *≤ 0.05 domestic exposure versus double exposure

^e^*P *≤ 0.05 domestic exposure versus unexposed

^f^*P *≤ 0.05 double exposure versus unexposed

^§^*P* comparisons between the 4 groups

The clinical characteristics of asthmatic individuals can be seen in Table 3. Compared to smokers and to unexposed persons, patients exposed to household firewood smoke showed poorer asthma control, greater disease severity and lung function impairment. Doubly exposed persons had a higher frequency of uncontrolled asthma, severe asthma and a history of hospitalization in intensive care units compared to the group without exposure or with any of the two isolated exposures. There was no significant difference between groups regarding the frequency of oral corticosteroids use, hospitalizations and use of emergency services.

**Table 3:** Clinical characteristics of the individuals with asthma (mild to moderate and severe) studied

Characteristics	Smokers (n = 78)	Household air pollution (n = 358)	Double exposure (n = 155)	No exposure (n = 405)	*P* ^§^
	n (%)	n (%)	n (%)	n (%)	
Asthma control status					
Uncontrolled	22 (28.2)	108 (30.2)^e^	52 (33.5)^f^	76 (18.8)	< 0.001
Controlled	56 (71.8)	250 (69.8)	103 (66.5)	329 (81.2)	
Asthma severity					
Mild to moderate	44 (56.4)^b.c^	124 (34.6)^d.e^	39 (25.2)^f^	245 (60.5)	< 0.001
Severe	34 (43.6)	234 (65.4)	116 (74.8)	160 (39.5)	
Oral corticosteroid bursts in the last 12 months					
None	48 (61.5)	192 (53.6)	82 (52.9)	239 (59.0)	0.329
1 or more	30 (38.5)	163 (45.5)	70 (45.2)	163 (40.2)	
No information	–	03 (0.8)	03 (1.9)	03 (0.7)	
Hospitalization for asthma last 12 months					
None	77 (98.7)	348 (97.2)	146 (94.2)^f^	397 (98.0)	0.079
1 or more	01 (1.3)	10 (2.8)	09 (5.8)	08 (2.0)	
Emergency visits for asthma in last 12 months					
None	55 (70.5)	244 (68.2)	103 (66.5)	249 (61.5)	0.194
1 or more	23 (29.5)	114 (31.8)	52 (33.5)	155 (38.3)	
No information	–	–	–	01 (0.2)	
ICU hospitalization during lifetime	07 (9.0)	45 (12.6)^e^	23 (14.8)^f^	24 (5.9)	0.003
Body mass index ≥ 30 kg/m^2^	27 (34.6)	122 (34.1)	51 (32.9)	123 (30.4)	0.691
First asthma symptoms after 18 years of age	20 (25.6)	124 (34.6)^e^	56 (36.1)^f^	97 (24.0)	0.002

^a^*P *≤ 0.05 smokers versus unexposed

^b^*P *≤ 0.05 smokers versus domestic pollution

^c^*P *≤ 0.05 smokers versus double exposure

^d^*P *≤ 0.05 domestic pollution versus double exposure

^e^*P *≤ 0.05 domestic exposure versus unexposed

^f^*P *≤ 0.05 double exposure versus unexposed

^§^*P* comparisons for the 4 groups

The spirometric measurements of our sample are displayed in Table 4. The dual-exposure group had worse lung function and evidence of fixed airway obstruction in a larger proportion than found in the other groups. A statistically significant difference was observed in the spirometric parameters between the groups. The dual-exposure group had lower spirometric flow measurements and a higher proportion of subjects with criterion for Chronic Obstructive Pulmonary Disease (COPD), namely a FEV_1_/FVC ratio < 70% after bronchodilator use. Reversibility in response to FEV_1_ post-bronchodilator was also more frequent in the double-exposure patients (47 cases, 31.3%); this proportion was followed by that of unexposed asthmatics (108 cases, 27.1%), then by the group exposed only to household pollution (93 cases, 26.4%), finally by the group exposed only to tobacco (20 cases, 25.6%).

**Table 4:** Spirometric parameters of the individuals with asthma (mild to moderate and severe) studied

Characteristics	Smokers (n = 78)	Household pollution (n = 358)	Double exposure (n = 155)	No exposure (n = 405)	P^§^
Pre-BD*					
FEV_1_	75 ± 18^b.c^	70 ± 19^d.e^	66 ± 17^f^	74 ± 18	< 0.001
FEV_1_/FVC ^††^	0.73 ± 0.12	0.71 ± 0.13^e^	0.71 ± 0.14^f^	0.73 ± 0.13	0.073
FEF_25–75 %_	64 ± 34^c^	57 ± 35^d.e^	46 ± 27^f^	65 ± 32	< 0.001
Post-BD*					
FEV_1_	82 ± 17^b.c^	76 ± 18^d.e^	72 ± 16^f^	80 ± 17	< 0.001
FEV_1_/FVC^††^	0.76 ± 0.12^c^	0.74 ± 0.12^e^	0.72 ± 0.13^f^	0.77 ± 0.13	0.001
FEF_25–75 %_	76 ± 36^b.c^	66 ± 38^d.e^	52 ± 28^f^	77 ± 34	< 0.001
FEV_1_ post-BD < 80%^†^	28 (35.9)^b.c^	203 (56.7)^d.e^	101 (65.2)^f^	180 (44.4)	< 0.001
FEV_1_/FVC post-BD < 70%^†;††^	25 (32.1)	127 (35.5)^e^	60 (38.7)^f^	111 (27.4)	0.036
FEF _25–75 %_ pre-BD < 60%^†^	41 (52.6)^c^	221 (61.7)^d.e^	110 (71.0)^f^	191 (47.2)	< 0.001

Values expressed as mean ± std. dev. except where otherwise indicated

^a^*P *≤ 0.05 smokers versus unexposed

^b^*P *≤ 0.05 smokers versus domestic pollution

^c^*P *≤ 0.05 smokers versus double exposure

^d^*P *≤ 0.05 domestic pollution versus double exposure

^e^*P *≤ 0.05 domestic pollution versus unexposed

^f^*P *≤ 0.05 double exposure versus unexposed

* BD–bronchodilator

^†^Values expressed as n (%) except where otherwise indicated

^††^Absolute values

^§^*P* comparisons between 4 groups

The variables associated with the control and severity of asthma were included in the multivariate analysis (Table 5). It was verified that the isolated exposure household pollution and double exposure to smoking and household pollution were risk factors related to control and the severity of asthma. The potential confounders that were considered are shown in Table 5.

**Table 5:** Multivariate analysis of factors possibly associated with lack of control and severity of asthma among the individuals studied

Factors	Control			Severity		
	β	OR	95% IC	β	OR	95% IC
Age	0.010	1.010	0.999–1.021	0.079	1.082	1.069– 1.096
Feminine gender	0.428	1.534	1.017–2.315	–	–	–
Exposure to tobacco	0.759	2.136	1.198–3.809	–	–	–
Exposure to household pollution	0.561	1.753	1.202–2.557	0.377	1.457	1.038–2.046
Doubly exposed to smoking and household pollution	0.709	2.031	1.260–3.273	0.461	1.586	0.980–2.567

Adjusted for age in years, gender, exposure only to cigarette smoking, exposure only to household pollution, doubly exposed to smoking and household pollution, relation cotinine/creatinine ratio (≥ 196.98 μg/g)

## Discussion

The results of this cross-sectional study demonstrate exposure to household air pollution and smoking are common in adults with asthma in Salvador, Brazil; we also found that dual exposure is associated with worse spirometric parameters, worse disease control and greater severity of asthma, indicating the harmful effect of these exposures. The findings of this study suggest that individuals with asthma who reside in households that use wood burning have a significantly greater risk for lack of asthma control (OR 1.75, 95% CI 1.20–2.56) and greater severity (OR 1.46, 95% CI 1.04–2.05), as well as the combined effects of exposure to biomass and tobacco smoke on the risk were greater for the lack of control (OR 2.03; 95% CI 1.26–3.27) and asthma severity (OR 1.59; 95% CI 0.98–2.57).

The burden of exposure to household pollution was greater than the load of exposure to smoking; nevertheless, among Brazilian urban populations that do not burn firewood for cooking or heating, this epidemiological background is often neglected because it usually does not occur while living in the City, but while they lived in the countryside in the past. In this study, only 21 (2.1%) individuals declared themselves to be current smokers, a proportion lower than that found among Brazilian adults according to data from the Surveillance of Risk Factors and Protection for Non-Communicable Chronic Diseases (VIGITEL) [32]. We are not aware of studies that have evaluated the association between exposure to household air pollution and asthma severity in Brazil.

The smoke resulting from wood combustion includes a complex mixture of different chemicals, such as fine particles (PM 10 and PM 2.5), carbon monoxide, free radicals and other respiratory irritants [33]. Cigarette combustion produces more than 7000 different compounds [34]. These pollutants, through different mechanisms, contribute to the inception or aggravation of airway inflammation [33, 35], and may contribute to the increased risk of asthma in exposed adults [36]. The same mechanisms may contribute to risk of asthma severity and may start in childhood.

Exposure to household pollution had high frequency (36.0%) in our sample, followed by a combination of household pollution and smoking (15.6%). The main source of wood burning is the use of the wood stove, a common practice for individuals in rural areas [37–39]. We observed an association between longer time of residence in the rural area and lower family income. Because the state of Bahia is in a tropical region (Latitude of Salvador: 13°S), firewood combustion is not used to heat homes. But eventually poverty can lead to the use of this fuel for food cooking and lighting even in the City, and thereby contributes to the occurrence of worse clinical outcomes among exposed individuals. We supposed exposure to second hand cigarette smoke in the workplace or at home might be a confounder of asthma outcomes and cotinine measurements and decided to record and analyse it. But it does not seem to be the case in this study.

We acknowledge information related to the use of wood stoves such as frequency of use, type and condition of the equipment, ventilation and size of the home should be taken into account. They influence the concentrations of the pollutants in the environment and, consequently, condition the risk for respiratory health [40], and recognise lack of these information are a limitation of our study. The reason we did not plan on collecting these indicators of degree of exposure was two fold: (i) this happened mostly in the past; (ii) it was not a primary objective of our study. “Subjects are classified as having severe asthma according to the status they were enrolled in ProAR Severe Asthma Cohort, on average many years ago. We acknowledge the definition of severe asthma most widely used nowadays (ATS/ERS Task Force. ERJ 2014) does not apply to many of them. Now some of these subjects have their asthma well controlled or partially controlled by proper treatment. Asthma severity and control reflect different aspects of the disease, in such manner someone may have severe asthma well controlled with high dose of medications. Several studies related to our cohort have documented the impact of our specialized multidisciplinary care, free of charge, on health resources utilization, including emergency visits and hospitalizations. We believe the combination of free medication and meticulous follow up by a multidisciplinary team combined with patient education for use of an action plan have made a major difference.”

A cross-sectional study conducted with 397 children in a rural community in Montana (USA) found an association between exposure to second hand cigarette smoke and asthma symptoms, as opposed to home household air pollution, which presented no association [41]. In contrast, a cross-sectional study by Kraai et al. [42] conducted with 630 children living in rural settings in Venezuela, found an association between both household exposure to wood smoke and second hand cigarette smoke (more than 10 cigarettes per day) and a higher risk of asthma symptoms.

It is recognized that exposure to cigarette smoke is associated with accelerated decline in lung function, increased severity of asthma, and use of health services [7, 12]. In the group exposed only to smoking, a higher frequency of current smoking and secondary exposure to cigarette smoke was observed. The amount of cigarettes smoked is related to the risk of lack of control of asthma [43], while second hand tobacco smoke is an important constituent of indoor air pollution and is also associated with the risk of asthma, disease exacerbation, compromised pulmonary function, absence of asthma control and greater severity of the disease [36, 44, 45]. There is evidence of the dose-dependent relationship between secondary exposure to cigarettes and severity of asthma [46]. The evaluation of exposure to cigarette smoke is commonly performed through the application of questionnaires. However, it is useful to validate self-reported smoking status in respiratory care settings, because patients may omit information. In this context, the use of biomarkers is essential: dosing of cotinine, a nicotine metabolite is regarded as a gold standard biomarker because of its high accuracy [47, 48]. All the participants evaluated in our study were submitted to biochemical confirmation of the smoking status through the measurement of the cotinine/urinary creatinine ratio. In our sample, a higher frequency of individuals with high urinary cotinine levels was observed in the dual-exposure group, which reflects higher intensity of this exposure in this group and is in agreement with the data obtained by the application of a questionnaire. The apparent discrepancy between the exclusion criteria of subjects with a history of smoking > 10 pack years and the observation of various subjects found to have a smoking load over this limit in the study, is likely due to the difference in quick routine clinical questions and a meticulous research interview.

The effects of these exposures on severity and poorer control of asthma are recognized, but some studies have reported only the relationship of unfavourable outcomes with exposure to smoking, with no significant impact associated with exposure to household pollution [5, 49]. In our sample, we found that exposure to household pollution was associated with lack of control and severity in asthmatic adults. In the dual-exposure group, we noted a higher frequency of severe asthma, worse control of the disease and a higher frequency of hospitalizations in intensive care units for asthma at some time in life. All of these confirm the fact that exposure to smoking combined with household pollution constitute the most serious scenario and has to be seriously regarded as a high risk situation. Confirming previous information, the dual exposure group also presented greater intensity of airway obstruction measured by spirometry (through FEV_1_, FEV_1_/FVC and FEF_25–75%_ and fixed airway obstruction). Few studies have examined the association of these exposures on lung function in adults with asthma and their findings are contradictory. Exposure to cigarette smoke has been strongly associated with impairment of lung function in adults with asthma [6, 50, 51]. In our study, the doubly exposed group presented greater airflow limitation in relation to the other groups, and the reduction in FEF_25–75%_ is more pronounced than the reduction in FEV_1_ or FEV_1_/FVC, which suggests predominant obstruction of the small airways, confirming what has been just reported by Jetmalani et al. [52]. In a study of 3471 individuals aged 18–69 years, Hersoug et al. [49] found that individuals exposed for more than 5 h a day to cigarettes were at a higher risk of experiencing a decline in lung function (FEV_1_), suggesting a dose-dependent effect of the exposure. In a prospective cohort study with adult subjects, it was found that the effects of smoking on lung function differed between men and women, with lung function recovering more rapidly in women; however, current female smokers with airway obstruction had a greater decline in lung function compared to men [50]. Smoking is associated with increased bronchial hyperreactivity and is a risk factor for airflow obstruction. Exposure to second hand cigarette smoke, or the dual exposure also leads to impairment of lung function [7, 39].

The effects of exposure to biomass combustion products on lung function are widely known [39, 53, 54]. However, evidence of the effects of this exposure on lung function in adults with asthma is controversial. A population-based cross-sectional study conducted in adults in Denmark did not identify that the use of wood stoves was associated with reduced lung function among asthmatics [49]. In contrast, a study carried out in India, reports that exposure to biomass fuel among non-smoking asthmatics reduces pulmonary function parameters [55].

The mechanisms that cause decline in lung function in asthmatics exposed to smoking and household pollution are unclear. There is evidence that individuals exposed to both household pollution and smoking present lung damage characterized by increased inflammation, structural changes in the lung parenchyma, and remodelling of the airways [56, 57]. The combined exposure of pollutants associated with increased exposure load may have contributed to the greater decline in lung function in the dual exposure group. Our findings provide further evidence corroborating previous reports of the harmful effects of each of these exposures on the symptoms of lung disease and function in adults with asthma, bringing novel evidence the problem of household pollution is more relevant than smoking in our setting and dual exposure is associated with worse outcomes than each single exposure taken in isolation.

Among the potential limitations of our study, besides the lack of precise measurements of the intensity of exposure to household pollution, we shall consider the possibility of selection bias of individuals with severe asthma in relation to smoking. We excluded subjects with severe asthma who had previously reported a history of smoking load of more than 10 pack/years at the time of admission or during follow up at ProAR reference clinic. At the study visit interview some patients reported a smoking load greater than 10 pack/years and were not excluded at this time point. Therefore, the low proportion of smokers in our sample may be related to a clear selection bias. It does not interfere with the internal and external validity of our findings, however. A third limitation resides in the fact that our study is not completely population-based and our sample was not obtained randomly. However, considering that ProAR is the main reference centre for severe asthma in Salvador, our sample is the most representative we could obtain. On the other hand, the sample of patients with mild to moderate asthma was obtained somewhat randomly from the population of users of the Unified Health System of Salvador, the same population from which the cases of severe asthma originate.

## Conclusions

In this study on adults with asthma we report evidence of the harmful additive effects of smoking and household pollution, which are associated with worse outcomes than each individual exposure. The dual-exposure was significantly related to poorer asthma control, greater severity of asthma, and worse lung function. We also observed that exposure to household pollution was more frequent and was associated with worse outcomes than smoking in our sample of urban dwellers, made up in part by rural migrants.

## Abbreviations

COPD: chronic obstructive pulmonary disease
ANVISA: National Agency of Sanitary Surveillance
IBGE: Brazilian Institute of Geography and Statistics
GINA: Global Initiative for Asthma
ProAR: Program for Asthma Control in Bahia
SUS: Unified Health System
ACQ-6: Asthma Control Questionnaire in six-questions version
ATS/ERS: American Thoracic Society/European Respiratory Society
FEV_1_: forced expiratory volume in the first second
FEV_1_/FVC: ratio between FEV_1_ and FVC (forced vital capacity)
FEF_25–75%_: forced expiratory flow 25–75%
SPSS: Statistical Package Software for Social Science
VIGITEL: Surveillance of Risk Factors and Protection for Noncommunicable Chronic Diseases
